# Supplementary material for: Production of antigenically stable enterovirus A71 virus-like particles in Pichia pastoris as a vaccine candidate
Source: J Gen Virol. 2023 Jun 30;104(6):001867. doi: 10.1099/jgv.0.001867 (PMC10773253; doi:10.1099/jgv.0.001867)
Supplement: Supplementary material 1 [file jgv-104-1867-s001.pdf]

Supplementary material:

Figure S1:

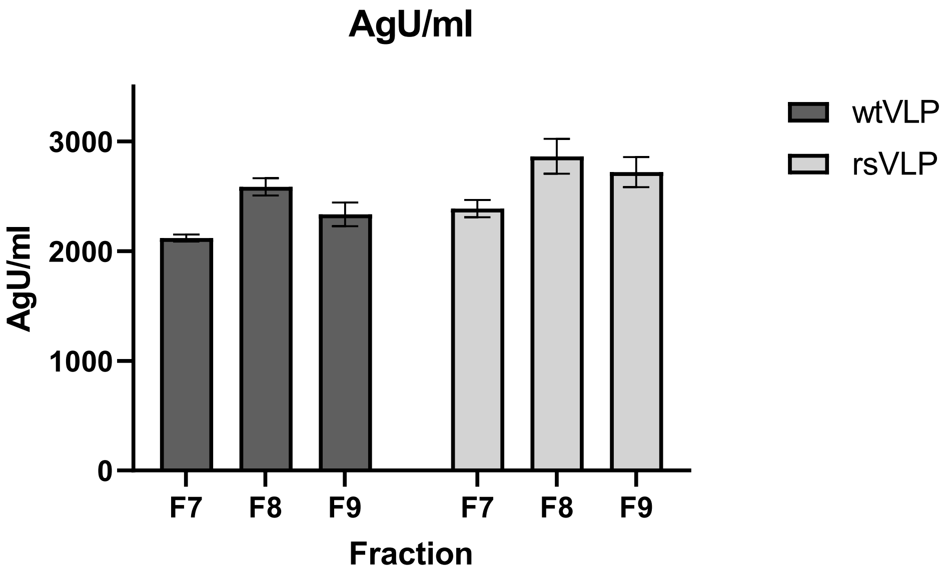

Figure S1: Antigen concentration (AgU/ml) of peak VLP fractions determined by the CT11F9 antibody relative to the 18/116 antigen standard.

Figure S2:

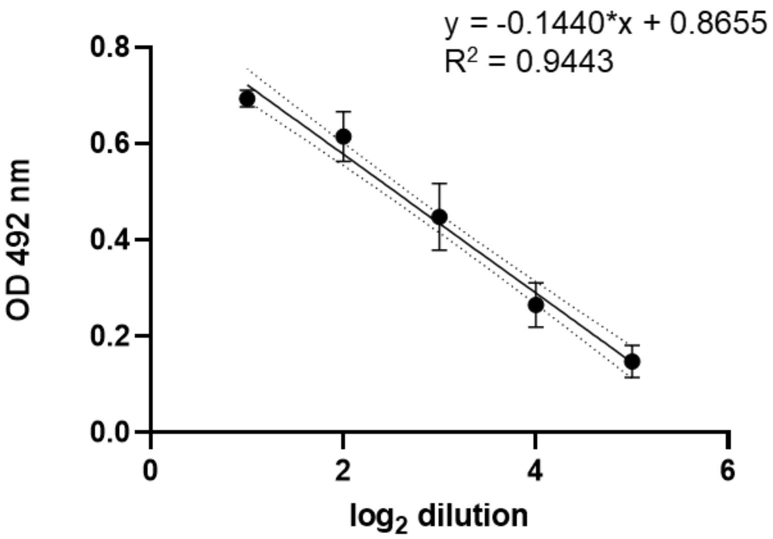

Figure S2: Relationship between OD and log_2 dilution of antigen, used to calculate the proportion of HAg in samples.

Figure S3:

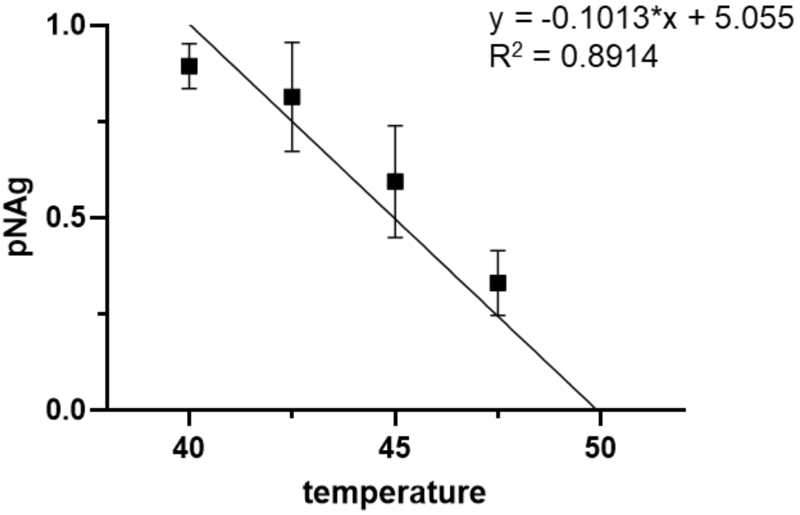

Figure S3: Linear regression to estimate antigenic conversion temperature of rsVLPs between 40°C and 50°C.

Figure S4:

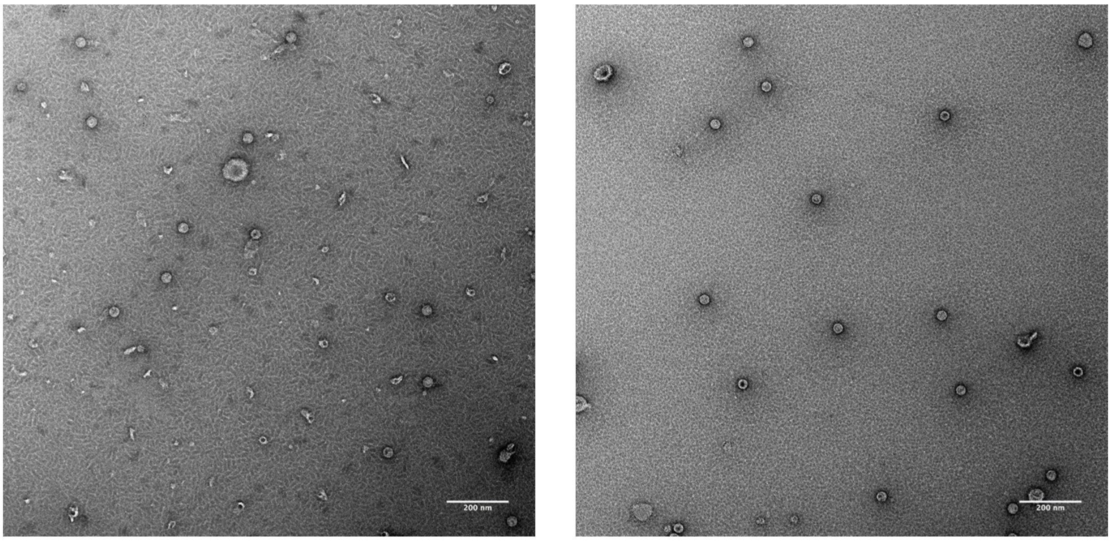

Figure S4: Negative stain TEM (using 2% UA) of samples of wtVLPs (left) and rsVLPs (right), scale bar = 200 nm.

Figure S5:

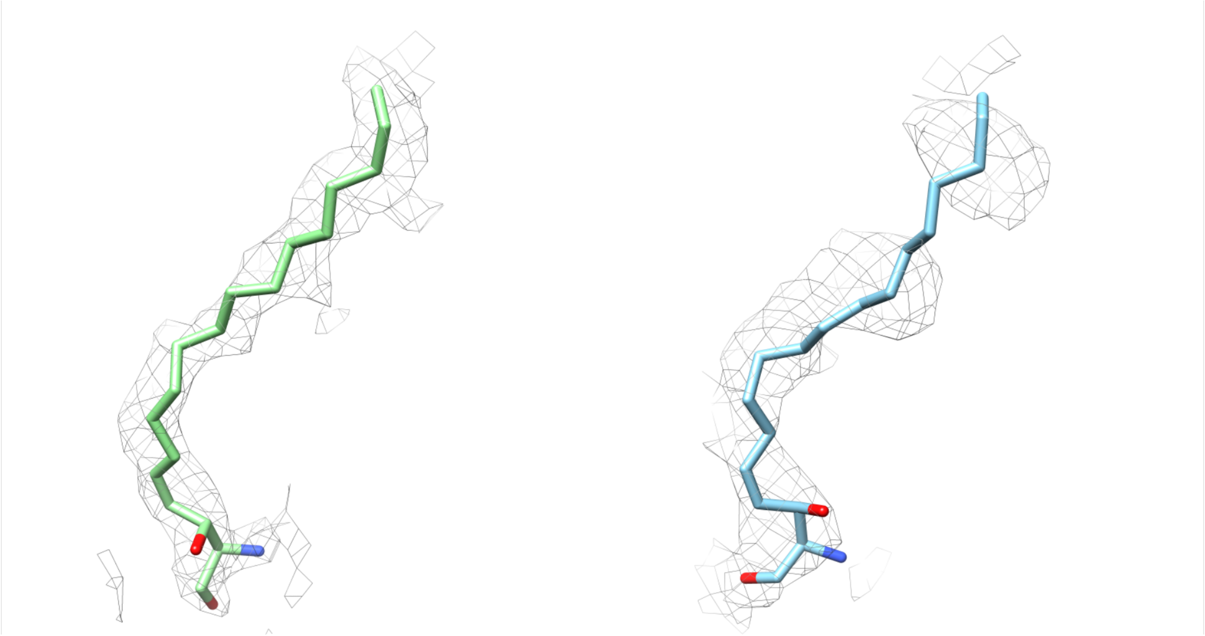

Figure S5: Density indicating occupancy of the pocket, present in the major population (left) and the minor population (right) shown at 2.5 rmsd.

Figure S6:

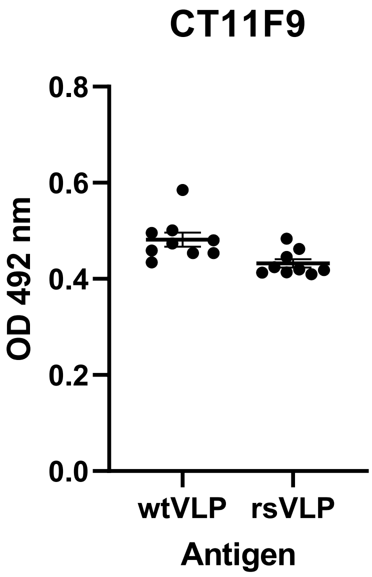

Figure S6: Reactivity of VLP (captured on ELISA plates) with CT11F9 antibody.

Table S1: specificity of the antibodies used to detect EVA71 antigenic states.

Antibody	Source	Specificity	Uses
CT11F9	mouse	NAg and HAg	ELISA
mAb 979	mouse	HAg	ELISA/western blot
16-2-2D scFv	human	NAg	ELISA

Table S2: Antigen composition of samples at a range of temperatures

$$proportion\ HAg = 2^{(OD\ 4°C\ -OD\ 55°C)/incline}$$

Temperature (°C)	pHAg rsVLP	pNAg rsVLP	pHAg wtVLP	pNAg wtVLP
**4**	0.13	0.87	1.11	-0.11
**30**	0.11	0.89	0.80	0.20
**35**	0.10	0.90	0.69	0.31
**37.5**	0.11	0.89	0.84	0.16
**40**	0.14	0.86	0.97	0.03
**42.5**	0.31	0.69	1.16	-0.16
**45**	0.46	0.54	1.18	-0.18
**47.5**	0.86	0.14	1.32	-0.32
**50**	1.10	-0.10	1.54	-0.54
**52.5**	1.07	-0.07	1.17	-0.17
**55**	1.00	0.00	1.00	0.00
**60**	0.80	0.20	0.73	0.27

Table S3:

	**rsVLP**
**Microscope**	FEI Titan Krios
**Detector mode**	Counting
**Camera**	Falcon IV
**Voltage (kV)**	300
**Pixel size (Å)**	0.91
**Nominal magnification**	130,000×
**Exposure time (s)**	5
**Total dose (e^-/Å^2)**	31
**Number of fractions**	42
**Defocus range (μm)**	–0.8 to –2.9
**Number of micrographs**	20,419
**Acquisition software**	Thermo Scientific EPU

Table S4:

**Model**	***rsVLP***
**EMDB ID**	EMD-16450
**PDB ID**	PDB-8C6D
**CryoEM map processing**	
Symmetry imposed	I1
Number of particles contributing to map	11,789
Map resolution (FSC = 0.143) (Å)	2.4
Map resolution range at atomic coordinates (Å)	2.4-2.8
Map sharpening B factor (Å^2)	-133.6
**Residues modelled**	A 58-297<br>B 13-45, 83-323<br>C 1-242
**RMSD**	
Bond lengths (Å)	0.0096
Bond angles (°)	1.33
**Validation**	
All-atom clashscore	8.17
MolProbity score	2.21
Rotamer outliers (%)	3.89
**Ramachandran plot**	
Favoured (%)	95.45
Allowed (%)	4.55
Outliers (%)	0.00
